# Structural and Functional Connectivity of Default Mode Network underlying the Cognitive Impairment in Late-onset Depression

**DOI:** 10.1038/srep37617

**Published:** 2016-11-25

**Authors:** Yingying Yin, Xiaofu He, Mingze Xu, Zhenghua Hou, Xiaopeng Song, Yuxiu Sui, Zhi Liu, Wenhao Jiang, Yingying Yue, Yuqun Zhang, Yijun Liu, Yonggui Yuan

**Affiliations:** 1 Department of Psychosomatics and Psychiatry, ZhongDa Hospital, School of Medicine, Southeast University, Nanjing 210009, P. R. China; 2 Institute of Psychosomatics, Medical School of Southeast University, Nanjing 210009, China; 3 Department of Psychiatry, Columbia University and New York State Psychiatric Institute, New York 10032, USA; 4 Department of Biomedical Engineering, Peking University, Beijing 100871, China; 5 Department of Psychiatry, Nanjing Medical University affiliated Nanjing Brain Hospital, Nanjing, China; 6 Shandong Provincial Key Laboratory of Wireless Communication Technologies, School of Information Science and Engineering, Shandong University, Jinan 250100, China; 7 Department of Psychiatry & McKnight Brain Institute, University of Florida, Gainesville, FL 32610, USA; 8 Department of Psychology, Southwest University, Chongqing 400715, China

## Abstract

To identify the association between the functional and structural changes of default mode network (DMN) underlying the cognitive impairment in Late-onset depression (LOD), 32 LOD patients and 39 normal controls were recruited and underwent resting-state fMRI, DTI scans, and cognitive assessments. Seed-based correlation analysis was conducted to explore the functional connectivity (FC) of the DMN. Deterministic tractography between FC-impaired regions was performed to examine the structural connectivity (SC). Partial correlation analyses were employed to evaluate the cognitive association of those altered FC and SC. Compared with controls, LOD patients showed decreased FC between DMN and the cingulo-opercular network (CON), as well as the thalamus. Decreased FA and increased RD of these fiber tracts connecting DMN with CON were found in LOD patient. The DMN-CON FC and the FA, RD of the fiber tracts were both significantly correlated with the cognitive performance. Therefore, the cognitive impairment in LOD might be associated with the decreased FC between the DMN and the CON, which probably resulted from the demyelination of the white matter.

Late-onset depression (LOD) is often characterized by cognitive impairment (CI) which brings a heavy burden of disability to patients^1^. Although LOD has a low prevalence, the number of patients is rising perpendicularly with the aggravated aging problem^2^. Patients with LOD were certified to have poor outcomes, including poor response to antidepressant treatment, greater illness severity, higher relapse rate, and greater duration of illness^3^. But even worse, LOD is considered as a clinical syndrome associated with an increased risk of developing Alzheimer’s disease due to the cognitive impairment^4^,^5^. Despite the notorious actuality, the cerebral malfunction underlying the cognitive impairment in LOD is still ambiguous.

Since the functional magnetic resonance imaging (fMRI) was applied to human brain research, the sophisticated functions of various brain regions have been fully studied. Resting state fMRI (R-fMRI) is an efficient technique to detect disease-related functional alterations before structural deformation has emerged^6^ and is quite preferred by virtue of convenient operation. During resting state, the default mode network (DMN) is a group of well-established areas involving in self-referential function^7^, as well as emotional and cognitive processing^8^,^9^. The posterior cingulate cortex (PCC), a key node of the DMN, was suggested to have an integrative role between emotion and episodic memory retrieval^10^. The dysfunction of the DMN has been widely reported in major depressive disorder. For instance, subregions such as subgenual cingulate and PCC in the DMN showed increased activity during emotional processing in depressive patients compared to healthy controls^11^,^12^,^13^. Normal aging is also associated with alterations in the activity and connectivity of brain regions within the DMN. Old adults showed decreased connectivity and ability to suppress low-frequency oscillations of the DMN. The strength of the functional connectivity (FC) of PCC with medial prefrontal cortex (mPFC) positively correlated with lower performance during the working memory task in older adults^9^. Accordingly, it is possible that the disruption of FC within the DMN may underlie the cognitive impairment in LOD patients. Our previous works have found the abnormal FC of DMN and its contribution to the cognitive impairment in remitted geriatric depressive patients^14^,^15^,^16^. In this study, we will focus on the DMN connective pattern of LOD patients.

Vascular risk factors of LOD have been well described (e.g., cardiovascular disease, stroke, hypertension, and diabetes), which may lead to demyelination of the white matter tract^17^. In the early stage of the disease, region-specific synapse loss, neurite retraction, and gliosis precede neuron loss (Brun *et al.*, 1995) causing neural system dysfunction and symptoms that may anticipate MRI-detectable atrophy. By measuring the diffusion of water in biological tissue, diffusion tensor imaging (DTI) could determine the microstructural abnormalities of the white matter tract^18^. Conjoint multiple diffusion tensor measures such as fractional anisotropy (FA), mean diffusivity (MD), axial diffusivity (AD) and radial diffusivity (RD) is an efficient approach for parsing the injury type of the white matter^19^. For instance, reductions in FA accompanied by increased RD but no change in AD may represent demyelination^20^,^21^, but axonal degeneration is characterized by reductions in FA accompanied by increases in RD and decreases in AD^22^, edema will decrease FA but increase MD, and neoplasia may decrease the MD^23^. Hitherto, numerous researches attest the white matter injury in late-life depression using DTI^17^,^24^,^25^. While several studies have focused on the relationship between FC and structural connectivity (SC) in healthy volunteers^26^,^27^, few studies have explored this relationship in LOD patients. Although Sexton *et al.* examined grey matter, white matter, and FC concurrently in late-life depression, they did not integrate these diverse modalities well with a reliable procedure^19^. In the healthy volunteers, a significant association between the FC and SC in the DMN has been found using FC analysis in conjunction with white matter tractography^28^. LOD patients who generally have higher vascular disease risk, the association between the FC and SC is plausibly more detectable.

In summary, previous investigations have separately demonstrated the disruption of the FC and the integrity of the white matter tracts of the DMN, and the correlation between them and the cognitive impairment in late life depression. But whether the altered FC resulted from the injury of white matter tracts is still unclear, especially in LOD which has a higher vascular risk. Therefore, in the present study, R-fMRI and DTI techniques were combined to determine the association between the functional changes and structural lesions of the DMN in LOD patients. We performed deterministic tractography analysis using DTI data based on the results of the FC analyses, which directly combined the FC and SC and brought us an intuitional understanding of the neuro-mechanism. We hypothesized that the cognitive impairment of LOD patients should be correlated to the altered FC in the DMN, that should be associated with the microstructural changes of white matter fiber.

## Materials and Methods

### Participants

This study was conducted with approval from the medical ethics committee for clinical research of ZhongDa Hospital affiliated to Southeast University. The methods were performed in accordance with approved guidelines. All participants were right-handed and gave informed consent to participate in this study. A total of 32 LOD inpatients from Nanjing Medical University affiliated Nanjing Brain Hospital and 39 well-matched normal controls (NCs) by advertising within the community were recruited during February 2009 to August 2010.

LOD patients were carefully screened in a semi-structured interview by two trained senior psychiatrists (Yonggui Yuan and Zhenghua Hou) and met the DSM-IV criteria for major depressive disorder currently. Only patients who had experienced their first depressive episode at the age of 55 or older and scored higher than 17 on the Hamilton Depression Rating Scale (HAMD_17_) were included. Exclusion criteria for LOD patients included the following: (1) Serious physical ailments, primary neurological illness, organic brain disease (e.g. former stroke, cerebral vascular malformations, or epilepsy), former brain injury; (2) another major psychiatric illness; (3) current or long-term alcohol or drug dependence; (4) dementia based on semi-structured interview with the patient according to DSM-IV and Mini-Mental State Examination (MMSE) ≤ 25^29^; (5) hypertension, diabetes, thyroid dysfunction, smoking and contraindications to MRI scanning and (6) antidepressant treatment within 6 months prior to the beginning of the study or previous psychiatric therapy. The clinical assessment for NCs was performed by an experienced psychiatrist to verify inclusion and exclusion criteria. Blood pressure and pulse were recorded, and more detailed physical examinations such as electrocardiograms and glucose levels were also performed. All NCs were physically healthy, with no hypertension, diabetes mellitus, cigarette smoking, history of severe illness and first-degree family history of psychiatric illness. They were required to have an HAMD score lower than 8 points and to have met the exclusion criteria 3, 4 and 5. All participants were right-handed, had no contraindications to MRI scanning, and had no cardiac or pulmonary disease that could influence the BOLD response.

### Cognition measurements

All subjects underwent a neuropsychological battery that consisted of overall cognition (MMSE), episodic memory (Auditory Verbal Learning Test, AVLT), working memory (Digit Span Test, DST), semantic memory (Verbal Fluency Test, VFT), perceptual speed (Trail Making Test, TMT-A) and executive speed (TMT-B, and Symbol Digit Modalities Test, SDMT). All neuropsychological scale scores were transformed to standard z values to avoid the influence of the different measurement units. Clinical and neuropsychological assessments were conducted at the same day of MRI scanning.

### MRI data acquisition

All the participants underwent MRI in the Department of Clinical Magnetic Resonance Imaging at Nanjing Brain Hospital affiliated of Nanjing Medical University using a Siemens 3.0 Tesla scanner with a 12-channel head coil. High-resolution 3-dimensional T1-weighted MRIs were acquired using a high-resolution spoiled gradient-recalled echo 3D axial images (repetition time (TR)/echo time (TE) = 2530 ms/3.34 ms; flip angle (FA) = 7°; acquisition matrix = 512 × 512; field of view (FOV) = 256 × 256 mm^2^; thickness = 1.33 mm). Whole-brain DTI was performed using an SE-EPI sequence, (TR = 9000 ms, TE = 104 ms, FOV = 230 mm × 230 mm, thickness = 2.5 mm; 64 gradient directions). Whole brain R-fMRI was performed using a gradient-recalled echo-planar imaging pulse sequence (30 axial slices, TR = 3000 ms; TE = 30 ms; FA = 90°; acquisition matrix = 64 × 64; FOV = 240 × 240 mm^2^; thickness = 4.0 mm; gap = 0 mm and 3.75 mm × 3.75 mm in-plane resolution parallel to the anterior commissure–posterior commissure line). This acquisition sequence generated 140 volumes in 7 min and 6 s. During R-fMRI, participants were instructed to lie still in the scanner, keep their eyes open, and refrain from falling asleep.

### Functional image preprocessing

All the image data were manually checked by two experienced radiologists for quality controls, and artifacts were removed before preprocessing.

R-fMRI image preprocessing was performed using the DPARSF software^30^. The first 10 time points were discarded for scanner calibration and for subjects to get used to the circumstance. The remaining 130 time points were corrected for timing differences between slices and for motion effects (six-parameter rigid body) using a reference volume in the center of the run. Participants with head motion more than 2.5 mm of the translational (x, y or z) or 2.5 degrees of rotational (α, β, γ) movement were ruled out for the next processing (6 LOD patients and 3 NCs). The structural image of each subject was coregistered to the functional image after head motion correction. Then the coregistered structural images were segmented using the unified segmentation algorithm which could improve the accuracy of spatial normalization significantly^31^ and then transformed into standard Montreal Neurological Institute (MNI) space. The functional images were also spatially normalized to MNI space by applying the parameters of structural image normalization and were resampled to a voxel size of 3 × 3 × 3 mm^3^. Subsequently, spatial smoothing was conducted with a Gaussian kernel of 6 mm full-width at half maximum. Following this, temporal filtering (0.01 Hz < f < 0.08 Hz) was applied to the time series of each voxel to reduce the effect of low-frequency drifts and high-frequency noise. Any linear trend was then removed.

DTI imaging preprocessing was performed using FMRIB Software Library (FSL) v5.0^32^, following the steps: (1) correction for eddy current-induced distortions and subject movements by EDDY tool (part of FSL toolbox); (2) skull-stripping using the brain extraction tool (BET, also part of FSL toolbox); (3) calculating the diffusion tensor to create FA, AD and RD maps with FMRIB’s Diffusion Toolbox (FDT, also part of FSL). Data quality was assessed by visually checking the raw DW images, eddy current corrected DW images, and the color-encoded FA images^33^ to exclude participants who had motion-corrupted DTI data.

T1-weighted 3D images were also performed skull-stripping using BET. To improve brain extraction quality, we used B-S combined options to remove the eye and neck slices^34^.

### FC in DMN

FC analyses were performed with the R-fMRI Data Analysis Tool Kit (REST, http://resting-fmri)^35^ by using a seed region correlation approach. A spherical region of interest (ROI) (radius = 10 mm) centered at the given MNI coordinates [0, −52, 30] located in the posterior cingulate cortex/precuneus (PCC/Pcu) area was used as the seed for the FC analysis. The coordinates obtained from the results of the study performed by Shulman *et al.*^36^. It was chosen because it could reliably predict the location of areas exhibiting reduced activity in the human brain, as measured with either PET or fMRI, during the performance of a variety of cognitive tasks. And it has been widely used as the ROI in the DMN-analyzing studies^37^,^38^. For each subject, a seed reference time course was obtained by averaging the time series of all voxels in the ROI. Then Pearson’s correlation analysis was performed between the seed reference time course and time series of each voxel in the brain in a voxel-wise way, with the global signal, white matter signal, Cerebral Spinal Fluid signal and the 6 head motion parameters as nuisance covariates. The resultant correlation coefficients were transformed into z-scores using Fisher’s transformation so that their distribution could better satisfy normality^36^.

### Deterministic Tractography

To examine the SC of DMN, deterministic tractography were conducted to visualize the fiber tracts using Diffusion Toolkit v0.6 and TrackVis v0.6 (http://www.trackvis.org/). Brain regions which showed significantly changed FC with the PCC/Pcu-ROI were selected as the ROIs for the deterministic tractography analysis. Fibers were reconstructed using FACT algorithm (Fiber Assignment by Continuous Tracking, a widely used method in DTI tractography). The tracking was beginning from the center of the seed voxel and continued along the direction of its largest component of the diagonalized diffusion tensor. When the tracking entered the next voxel, the direction was changed according to the later one and was continued until a voxel with FA < 0.2 (considered as grey matter or cerebral spinal fluid) was reached or the turning angle between adjacent voxels was greater than 45 degrees. We used the so-called “exhaustive search approach” in which fiber tracking was first performed from all voxels to create the streamline maps in which one streamline represents one fiber bundle^39^. A spline filtering was then applied to smooth the tracts. Since the streamline maps were created in individual diffusion space, ROIs were registered to individual diffusion space using SPM8′s co-registration (local T1 to local DTI space) and normalization (MNI T1 to local T1 space) methods (http://www.fil.ion.ucl.ac.uk/spm/). Then only tracts connecting ROIs were selected to count fiber number (FN) and to calculate the average FA, AD and RD of all the voxels along these fibers. FN was normalized by the ROI volumes in the diffusion space.

To control the cortical atrophy influence on the white matter degeneration, the grey matter volumes of ROIs were also calculated. Grey matter volume was analyzed using VBM8 toolbox by default parameter settings (http://dbm.neuro.uni-jena.de/vbm/). Firstly, T1 image data were normalized to MNI standard space using a set of non-linear functions and were segmented into grey matter, white matter, and cerebrospinal fluid images using SPM8 prior probability templates. Since we used modulated methods, the resulting images represent grey matter volume. Secondly, mean values in ROIs were extracted from all normalized, segmented, and modulated grey matter images to present the grey matter volume of ROIs. Finally, the grey matter volumes of ROIs were forwarded to statistical analysis as covariance.

### Statistical analysis

All analyses showed in Table 1 were performed using SPSS 18.0 software (SPSS, Inc., Chicago, IL) and *P* < 0.05 was considered as significant discrepancy. Independent two-sample t-test and Chi-square tests were applied for demographic statistics. General linear model was used for cognition measurements, controlling age and education.

FC statistical analyses (26 LOD patients and 36 NCs) were performed using the REST Statistical Analysis module. One-sample t-tests using the spatial maps of FC were performed to determine the FC patterns of the DMN in each group. Two-sample t-tests were used to compare the FC of DMN between LOD patients and NCs, controlling age and education. AlphaSim correction based on Monte Carlo simulation algorithm was used to correct for multiple comparisons (single voxel P value = 0.01, FWHM = 6 mm, with 61 × 73 × 61 mm^3^ whole brain mask, which yielded a corrected threshold of P < 0.05, cluster sizes >1080 mm^3^) (http://afni.nimh.nih.gov/pub/dist/doc/manual/AlphaSim.pdf). To further identify the cognitive significance of the altered FC in DMN, the averaged FC strength of each significant region in LOD patients was extracted.

The FA, MD, AD, RD and FN of the white matter tracts between the LOD patients and the NCs were compared through a general linear model by SPSS 18.0 software, taking age, education and grey matter volume of the ROIs as the covariates.

Partial correlation analyses using SPSS 18.0 software were employed to examine relationships between FC, FA, MD, AD, RD, FN and cognitive scores, controlling the effects of age and education.

## Results

### Demographic and neuropsychological assessments

The demographic and neuropsychological characteristics are summarized in Table 1. There was no age, gender or educational differences between the two groups. Compared with NCs, LOD patients displayed comprehensive deficits in cognitive performance, including language, executive and memory functions.

### FC of DMN

#### Within-group FC pattern of DMN

**Figure 1:**
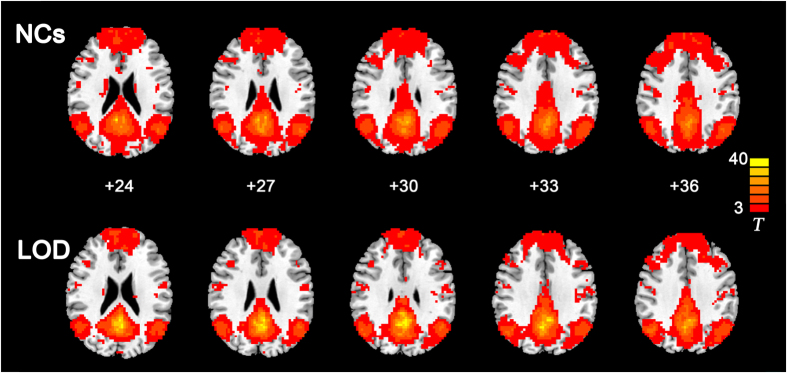
Functional connectivity patterns of the default mode network in LOD and NCs groups. AlphaSim correction based on Monte Carlo simulation algorithm was used to correct for multiple comparisons (single voxel P value = 0.01, FWHM = 6 mm, with 61 × 73 × 61 mm^3^ whole brain mask, which yielded a corrected threshold of P < 0.05, cluster sizes >1080 mm^3^). The color bar indicates the T value from one-sample t-test. Left in the figure indicates right side of the brain. Abbreviation: LOD, late-onset depression; NCs, normal controls.

In both of the two groups, strong positive correlations with the PCC/Pcu were observed in the medial prefrontal cortex (mPFC), bilateral angular gyrus, inferior temporal cortex, and medial temporal lobes in both groups, which correspond to the typical distribution of the DMN (Fig. 1).

#### Between-group different FC pattern of DMN

**Figure 2:**
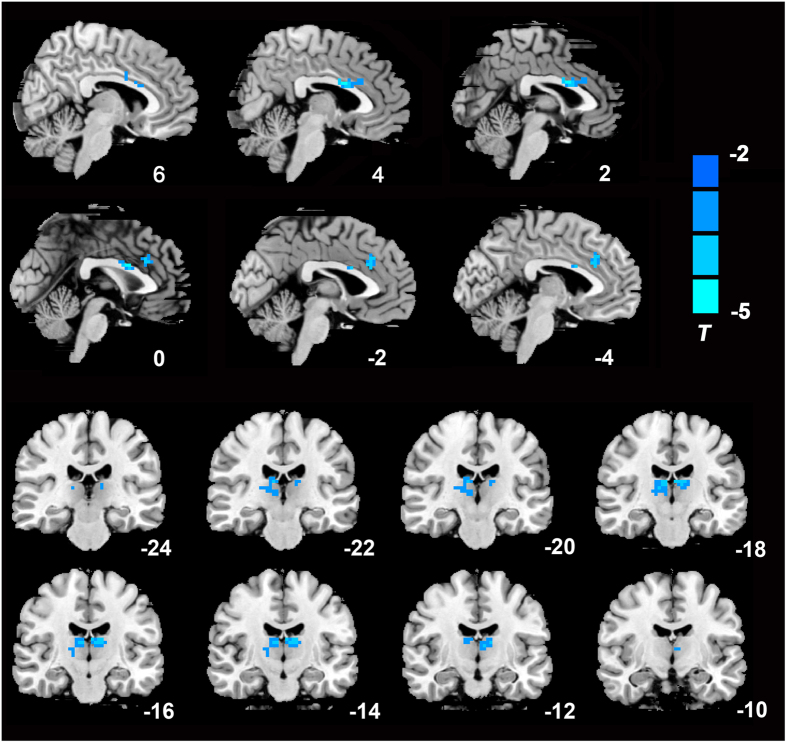
Significantly decreased functional connectivity maps of the default mode network in LOD patients. AlphaSim correction based on Monte Carlo simulation algorithm was used to correct for multiple comparisons (single voxel P value = 0.01, FWHM = 6 mm, with 61 × 73 × 61 mm^3^ whole brain mask, which yielded a corrected threshold of P < 0.05, cluster sizes >1080 mm^3^). Clusters located in the dACC and thalamus were shown on the sagittal and coronal brain image respectively. The color bar indicates the T value from two-sample t-test. Left in the figure indicates right side of the brain.

Compared with the NCs, LOD patients showed decreased FC between the PCC/Pcu and dorsal anterior cingulate (dACC) as well as the thalamus (Fig. 2).

### Deterministic Tractography

**Figure 3:**
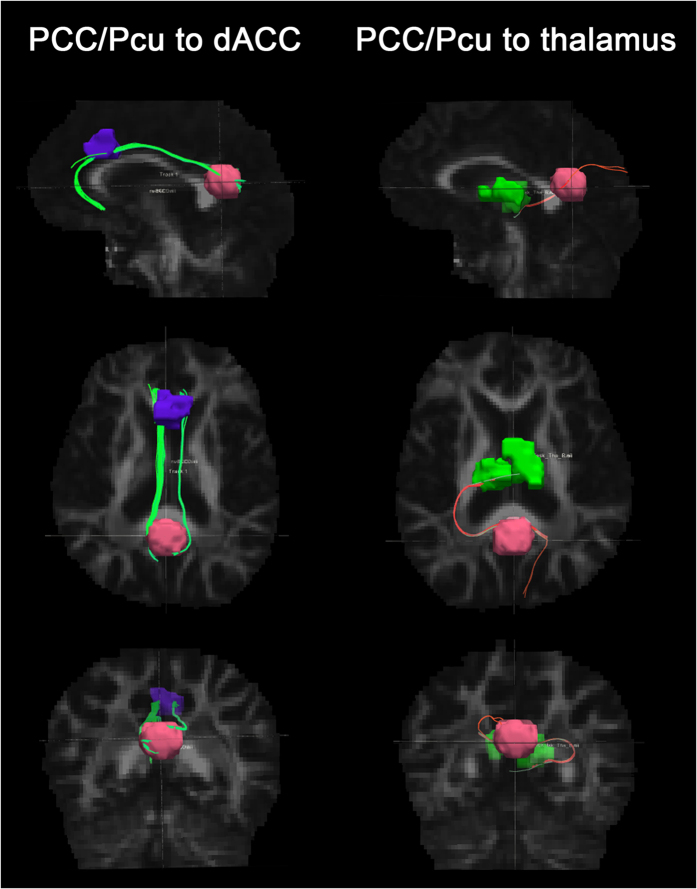
Deterministic tractography of brain regions which showed significantly changed FC with the PCC/Pcu (i.e. dACC and thalamus). The pink, purple, and green regions respectively displayed the ROI of PCC/Pcu, dACC and thalamus. The green fiber tracts are the cingulum bundles which connects PCC/Pcu and dACC. The pink fiber tracts are the thalamic fibers which connects PCC/Pcu and thalamus. Abbreviation: PCC/Pcu, posterior cingulate cortex/precuneus; dACC, dorsal anterior cingulated cortex; ROI, region of interest.

Decreased FA and increased RD of these fiber tracts connecting PCC/Pcu with dACC were found in LOD patients, without significant difference in MD, AD and FN. For the fiber tracts connecting PCC/Pcu with thalamus, there was no any discrepancy in FA, FN, MD, AD or RD between LOD and NCs groups (Fig. 3 and Table 2).

### Cognitive significance of FC and SC

The PCC/Pcu- dACC FC was significantly correlated with comprehensive cognitive performance. The FA and RD of the fiber tracts between PCC/Pcu and dACC respectively had positive and negative correlation with working memory and executive speed. The PCC/Pcu- thalamus FC was positively correlated with semantic memory and executive speed. But the fiber tracts between PCC/Pcu and thalamus did not show any correlation with the cognition measurements (Table 3).

## Discussion

In the present study, multimodal MRI was applied to explore the association between cognitive impairment in LOD and imaging alternation of DMN. In an attempt to testify the hypothesis that cognitive impairment of LOD patients should be correlated to the altered FC in the DMN which resulted from the microstructural injury of white matter fiber, we firstly determined brain regions that showed significantly altered FC of the DMN in LOD patients, then chose FC-impaired regions for further deterministic tractography to check the corresponding SC.

The most important finding of this work is that LOD patients showed decreased FC, FA and increased RD between PCC/Pcu and dACC but no significant change of AD and FN, indicating that the PCC/Pcu-dACC FC disruption may be resulted from the demyelination rather than degeneration or necrosis of the white matter. Notably, the FC and fasciculi’s FA between PCC/Pcu and dACC were both positively correlated with the working memory as well as executive speed, while negative correlation was found between these two cognitive scores and the RD of PCC/Pcu-dACC white matter tracts, which further provided evidences for the pathomechanism of the cognitive impairment in LOD. Although numerous works have suggested that cognitive impairment in late life depression stemmed from either FC disruption or SC (especially white matter) damage^14^,^15^,^16^,^17^,^24^,^25^,^39^,^40^,^41^,^42^, no one has merged the two modalities together. Sexton *et al.*^19^ have analyzed three modalities including FC, the integrity of white matter, and grey matter volume, but the three parts of the data analyses were mutually independent, and the patients they recruited were mostly remitted subjects. Therefore, they did not get any significant result. Our study avoided these deficiencies and merged the FC and SC analyses in a reliable way. Our findings provide a novel insight into the etiological research of LOD, i.e. the decreased FC of DMN underlying the cognitive impairment is probably stemmed from the demyelination of white matter. But it is regrettable that this FC-SC association was only discovered between PCC/Pcu and dACC. Even though the PCC/Pcu-thalamus FC was also decreased in LOD patients, there was no significant difference in the fiber tracts between the two areas. This phenomenon could be explained in the following ways. In terms of anatomy, the PCC/Pcu and dACC were directly connected by the cingulum bundles, but the fibrous connections between thalamus and PCC/Pcu were indirectly through thalamic radiations and superior longitudinal fasciculus^43^. In terms of function, dACC is an important hub of the dorsal nexus, which plays important roles in cognitions such as executive speed, working memory etc.^44^. It is also part of the cingulo-opercular network (CON) which is well-known to be involved in executive control functions such as conflict detection and decision-making^45^. Similarly, PCC is also important episodic memory retrieval^10^. The well-known cognition control center, dorsolateral prefrontal cortex probably works together with PCC via the bridge of dACC. Thus, our result suggested the cognitive impairment of LOD patients might refer to the destructive connectivity between DMN and CON rather than DMN itself. This finding was somewhat expanded our hypothesis. With regard to the thalamus, it is an inferior nerve center which primarily responsible for basic functions such as vasomotor activity, visceral sensations, hunger, thirst and so on^46^. Therefore, the associations between cognitive functions, FC, and SC, might be more prominent in higher order cortical areas than in lower order subcortical areas.

Inconsistent with previous findings, we did not detect any neuroradiological changes in a vital area of the DMN, the mPFC. A mass of studies have shown that the PCC/Pcu and the mPFC consistently decrease their activity in variety of cognitive tasks^47^,^48^. However, the findings of resting-state FC of DMN in depression were quite discrepant. While some studies found that depressed subjects had decreased resting-state FC^11^,^49^,^50^, other investigators reported increased resting-state FC^5^,^51^,^52^ in the DMN of depressive patients. The observed heterogeneity could be ascribed, in part, to differences in sample size, medications, and analysis methods, but it should be noted that resting-state DMN FC alterations related to depression and cognition could not be simply described with a pure increased or decreased model. A recent imaging study described a dissociation between the anterior and posterior parts of the DMN^6^. Our previous work also got similar results^39^ that opposite FC changes between anterior/posterior DMN and the cerebellums were found. In this study, the absence of FC changes in the mPFC may be principally explained by the population we studied (i.e. geriatric patients rather than young ones). Also since the mPFC mainly participates in modulating the emotion^53^, but the dominant symptoms of the geriatric patients are cognitive impairment rather than depression, the FC of mPFC might be less affected in our sample.

## Conclusion

In summary, according to the present results, the cognitive impairment in LOD, especially the executive speed and working memory are associated with the decreased FC between the DMN and the CON which may result from the demyelination of the white matter. These findings provide a new insight into the neural mechanism underlying the cognitive impairment in LOD patients. Our findings provided additional imaging evidence for the early diagnosis of LOD and prevent it from developing into dementia.

### Limitations

There are a few potential methodological limitations in the current study. First, the effects of physiological noise such as cardiac and respiratory pulsation during resting fMRI scans could not be completely eliminated^36^. Besides, spontaneous thoughts and random uncontrolled cognitive processing during rest state may influence the FC measures. Second, global signal regression may bias the correlation coefficients downward on average, but whether the anticorrelations introduced by global signal regression are artificial is still debatable to date^39^,^54^,^55^. Third, the tracked fibers depend on the accuracy of ROI. Moreover, the resolution of DTI data (1.8 × 1.8 × 2.5 mm^3^) is low, in which diffusion tensor model may not reflect the reality of a huge population of fibers in one voxel.

## Additional Information

**How to cite this article**: Yin, Y. *et al.* Structural and Functional Connectivity of Default Mode Network underlying the Cognitive Impairment in Late-onset Depression. *Sci. Rep.* **6**, 37617; doi: 10.1038/srep37617 (2016).

**Publisher's note:** Springer Nature remains neutral with regard to jurisdictional claims in published maps and institutional affiliations.

## Figures and Tables

**Table 1:** Demographic and neuropsychological data.

Items	LOD (n = 32)	NCs (n = 39)	χ^2^/t	*P*
Gender (male: female)^a^	11:21	19:20	1.482	0.223
Age (years)^b^	67.17 ± 5.83	65.40 ± 7.37	1.104	0.273
Education levels (years)^b^	9.31 ± 4.17	10.41 ± 3.10	−1.274	0.207
HAMD^b^	30.83 ± 4.58	2.42 ± 3.71	30.797	0.000*
Cognition measurements				
MMSE (overall cognition)^c^	28.50 ± 1.38	29.56 ± 0.77	−4.087	0.000*
AVLT (episodic memory)^c^	−0.74 ± 0.58	0.59 ± 0.85	−7.525	0.000*
VFT (semantic memory)^c^	−0.76 ± 0.64	0.62 ± 0.78	−8.030	0.000*
DST (working memory)^c^	−0.77 ± 0.67	0.61 ± 0.77	−7.961	0.000*
TMT-A (perceptual speed)^c^	0.62 ± 0.95	−0.50 ± 0.71	5.681	0.000*
TMT-B (executive speed)^c^	0.44 ± 0.97	−0.36 ± 0.86	3.681	0.000*
SDMT (executive speed)^c^	−0.88 ± 0.41	0.70 ± 0.73	−10.905	0.000*

Notes: a Chi square test; b Independent-samples t-test (data are presented as mean ± stand deviation); c General linear model, controlling age and education (data are presented as mean ± stand deviation); *P < 0.05.

Abbreviation: LOD, late-onset depression; NCs, normal controls; MMSE, Mini mental state exam; HAMD, Hamilton Depression Scale; AVLT, Auditory Verbal Learning Test; SDMT, Symbol digit modalities test; DST, Digit span test-forward and backward; VFT, Verbal fluency test-animal and verb; TMT-A, Trail making test-A; TMT- B, Trail making test-B.

**Table 2:** Deterministic tractography of the fiber tracts.

Items	LOD	NCs	F	*P*
PCC/Pcu to dACC	(N = 26)	(N = 29)		
FA	0.45 ± 0.06	0.48 ± 0.05	2.037	0.047*
FN (normalized)	0.02 ± 0.03	0.02 ± 0.02	0.011	0.992
MD ( × 10^−3^)	0.76 ± 0.09	0.72 ± 0.04	1.987	0.052
AD ( × 10^−3^)	1.15 ± 0.09	1.13 ± 0.05	1.022	0.311
RD ( × 10^−3^)	0.57 ± 0.11	0.51 ± 0.05	2.136	0.037*
PCC/Pcu to thalamus	(N = 9)	(N = 5)		
FA	0.54 ± 0.06	0.59 ± 0.08	1.460	0.170
FN (normalized)	0.004 ± 0.004	0.002 ± 0.002	0.740	0.474
MD (×10^−3^)	0.82 ± 0.07	0.77 ± 0.13	0.972	0.350
AD (×10^−3^)	1.35 ± 0.08	1.32 ± 0.09	0.666	0.518
RD (×10^−3^)	0.55 ± 0.08	0.49 ± 0.15	0.985	0.344

Notes: Statistical analysis using general linear model, taking the age, education and grey matter volume of regions of interesting as the covariates. (data are presented as mean ± stand deviation); *P < 0.05.

Abbreviation: LOD, late-onset depression; NCs, normal controls; PCC/Pcu, posterior cingulate cortex/precuneus; dACC, dorsal anterior cingulate; FA, Fractional anisotropy; FN, fiber number; MD, mean diffusivity; AD, axial diffusivity; RD, radial diffusivity.

**Table 3:** Correlation between functional/structural connectivity and cognition measurements.

Items	Statistics	MMSE	AVLT	VFT	DST	TMT-A	TMT-B	SDMT
PCC/Pcu to dACC								
FC	r	0.318	0.345*	0.362*	0.448*	−0.372*	−0.205	0.428*
(N = 62)	*P*	0.012	0.006	0.004	0.000	0.003	0.110	0.001
FA	r	0.256	0.065	0.258	0.345*	−0.027	−0.155	0.311*
(N = 55)	*P*	0.062	0.638	0.059	0.011	0.847	0.263	0.022
MD	r	−0.265	−0.174	−0.266	−0.208	0.038	0.056	−.277*
(N = 55)	*P*	0.053	0.209	0.051	0.131	0.784	0.687	0.042
AD	r	−0.092	−0.159	−0.156	−0.002	0.079	−0.008	−0.149
(N = 55)	*P*	0.508	0.251	0.261	0.990	0.572	0.955	0.282
RD	r	−0.304*	−0.157	−0.279*	−0.270*	0.015	0.076	−.296*
(N = 55)	*P*	0.025	0.258	0.041	0.048	0.913	0.583	0.030
FN	r	0.045	0.031	0.145	0.038	−0.120	0.049	−0.034
(N = 55)	*P*	0.749	0.822	0.297	0.787	0.386	0.728	0.809
PCC/Pcu to thalamus								
FC	r	0.200	0.249	0.306*	0.186	−0.235	−0.050	0.306*
(N = 62)	*P*	0.120	0.051	0.015	0.148	0.066	0.697	0.016
FA	r	0.238	0.163	0.425	0.489	−0.366	−0.333	0.544*
(N = 14)	*P*	0.413	0.577	0.130	0.076	0.198	0.244	0.044
MD	r	−0.175	−0.002	−0.285	−0.311	0.397	0.381	−0.457
(N = 14)	*P*	0.550	0.995	0.322	0.280	0.159	0.179	0.100
AD	r	−0.228	0.037	−0.075	0.031	0.347	0.271	−0.358
(N = 14)	*P*	0.434	0.899	0.799	0.916	0.224	0.349	0.209
RD	r	−0.140	−0.015	−0.333	−0.401	0.378	0.384	−0.449
(N = 14)	*P*	0.633	0.958	0.245	0.155	0.183	0.176	0.107
FN	r	−0.507	−0.169	−0.369	−0.154	0.265	0.112	−0.177
(N = 14)	*P*	0.064	0.565	0.194	0.600	0.360	0.703	0.544

Notes: P value was calculated by partial correlation analysis, taking age and education as the covariate; *P < 0.05.

Abbreviation: MMSE, Mini mental state exam; AVLT, Auditory Verbal Learning Test; DST, Digit span test-forward and backward; VFT, Verbal fluency test-animal and verb; TMT, Trail making test; SDMT, Symbol digit modalities test;. PCC/Pcu, posterior cingulate cortex/precuneus; dACC, dorsal anterior cingulate; FC, functional connectivity; FA, Fractional anisotropy; RD, radial diffusivity; MD, mean diffusivity; AD, axial diffusivity; FN, fiber number.
